# Blood sample collection in small laboratory animals

**DOI:** 10.4103/0976-500X.72350

**Published:** 2010

**Authors:** S Parasuraman, R Raveendran, R Kesavan

**Affiliations:** *Department of the Pharmacology, Jawaharlal Institute of Postgraduate Medical Education and Research, Pondicherry, India*

Collection of blood from small laboratory animals is necessary for a wide range of scientific research and there are a number of efficient methods available for that. It is important that blood sample collection from experimental animals should be least stressful because stress will affect the outcome of the study. Various regulatory agencies and guidelines have restricted the use of animals and the techniques used for blood collection in laboratory animals. This article deals with the approved blood collection techniques for laboratory animals like rodents, lagomorphs and nonrodents. Permission of the Institute Animal Ethics Committee has been obtained for the use of animals for demonstrating the techniques.

## GENERAL PRINCIPLES OF BLOOD COLLECTION IN ANIMALS

- The method of blood collection should be described in the protocol approved by the Institute animal ethics committee.
- It should be least painful and stressful. Blood sample may be collected under anesthesia [Table 1] or without anesthesia.[1]
- Adequate training is required for blood collection using any method in any species.
- In general, blood sample is withdrawn from venous, arterial blood vessels or heart chambers.
- Frequency of blood collection is important. Once in two weeks is ideal for nonrodents. If the study needs multiple blood samples, lagomorphs (e.g., hares and rabbit) can be used.
- All nonterminal blood collection without replacement of fluids is limited up to 10% of total circulating blood volume in healthy, normal, adult animals on a single occasion and collection may be repeated after 3 to 4 weeks. In case repeated blood samples are required at short intervals, a maximum of 0.6 ml/kg/day or 1.0% of an animal’s total blood volume can be removed every 24 hour.[2,3]
- If the study involves repeated blood sample collection, the samples can be withdrawn through a temporary cannula. This may reduce pain and stress in the experimental animals.
- The estimated blood volume in adult animals is 55 to 70 ml/kg body weight. Care should be taken for older and obese animals.[4] If blood collection volume exceeds more than 10% of total blood volume, fluid replacement may be required. Lactated Ringer’s solution (LRS) is recommended as the best fluid replacement by National Institutes of Health (NIH). If the volume of blood collection exceeds more than 30% of the total circulatory blood volume, adequate care should be taken so that the animal does not suffer from hypovolemia.[5]

**Table 1:** Commonly recommended anesthetic agents for laboratory animal experiments

Animal species	Short anesthesia	Medium anesthesia	Long anesthesia
Mice	Isoflurane (inhalation)	Xylazine + ketamine	Xylazine + ketamine
	Halothane (inhalation)	(5 mg + 100 mg i.m.)	(16 mg+60 mg i.m./i.p.)
		Xylazine + ketamine	Xylazine + ketamine
		(5 mg + 100 mg i.m.)	(16 mg +60 mg i.m./ i.p.) or
			Urethane (1200 mg/kg i.p.)
Guinea pig	Isoflurane (inhalation)	Xylazine + ketamine	Xylazine + ketamine
		(2 mg + 80 mg i.m.)	(4 mg + 100 mg i.m.)
Rabbits	Isoflurane (inhalation)	Xylazine + ketamine	Xylazine + ketamine
		(5 mg + 15 – 30 mg i.m.)	(5 mg + 100 mg i.m.)

Atropine (0.02 mg/kg s.c./i.m.) is used as a preanesthetic medication for all the species to reduce salivation, bronchial secretion and protect heart from vagal inhibition.[6–8]

## GENERAL METHODS FOR BLOOD COLLECTION

Blood samples are collected using the following techniques:[1]

- Blood collection not requiring anesthesia
  - Saphenous vein (rat, mice, guinea pig)
  - Dorsal pedal vein (rat, mice)
- Blood collection requiring anesthesia (local/general anesthesia)
  - Tail vein (rat, mice)
  - Tail snip (mice)
  - Orbital sinus (rat, mice)
  - Jugular vein (rat, mice)
  - Temporary cannula (rat, mice)
  - Blood vessel cannulation (rat, guinea pig, ferret)
  - Tarsal vein (guinea pig)
  - Marginal ear vein/artery (rabbit)
- Terminal procedure
  - Cardiac puncture (rat, mice, guinea pig, rabbit, ferret)
  - Orbital sinus (rat, mice)
  - Posterior vena cava (rat, mice)

## PROCEDURE FOR SAPHENOUS VEIN BLOOD SAMPLE COLLECTION[9]

Requirements include animal, rodent handling gloves, towel, cotton, sample collection tubes and 20G needle.

- Lateral saphenous vein is used for sampling while taking aseptic precautions.
- The back of the hind leg is shaved with electric trimmer until saphenous vein is visible. Hair removal cream can also be used.
- The animal is restrained manually or using a suitable animal restrainer.
- Hind leg is immobilized and slight pressure may be applied gently above the knee joint.
- The vein is punctured using a 20G needle and enough volume of blood is collected with a capillary tube or a syringe with a needle. The punctured site is compressed to stop the bleeding. While collecting blood:
  - the local anesthetic cream may be applied on the collection site
  - no more than three attempts are made
  - continuous sampling should be avoided and
  - collecting more than four samples in a day (24-hour period) is not advisable.

## PROCEDURE FOR DORSAL PEDAL VEIN BLOOD SAMPLE COLLECTION

Requirements include animal (rat or mice), rodent handling gloves, cotton, capillary tube, 23G/27G needle and blood sample collection tubes.

**Figure 1:**
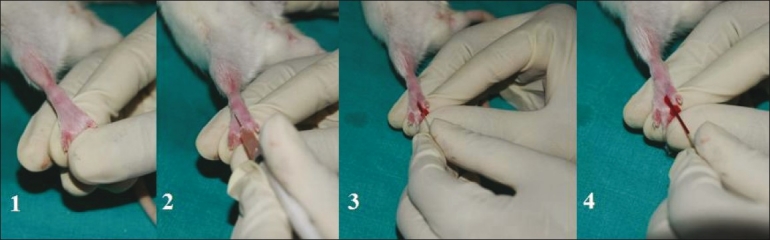
Blood sample collection from rat dorsal pedal vein

- The animal is kept in a restrainer.
- The hind foot around ankle is held and medial dorsal pedal vessel is located on top of the foot.
- The foot is cleaned with absolute alcohol and dorsal pedal vein is punctured with 23G/27G needle.
- Drops of blood that would appear on the skin surface are collected in a capillary tube and a little pressure is applied to stop the bleeding [Figure 1].

## PROCEDURE FOR TAIL VEIN BLOOD SAMPLE COLLECTION

Requirements include animal, rodent handling gloves, towel, cotton, sample collection tube and animal warming chamber.

**Figure 2:**
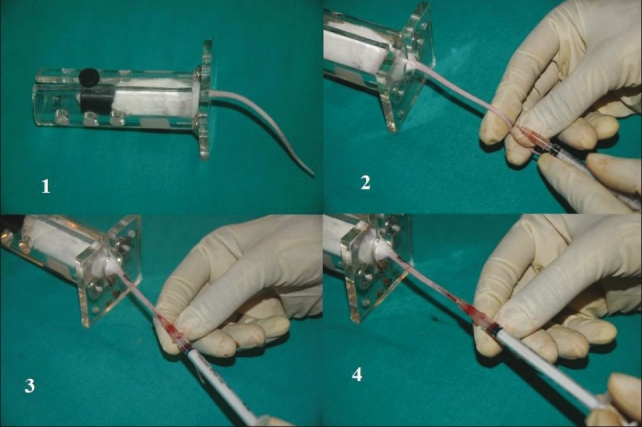
Blood sample collection from mouse tail vein

- This method is recommended for collecting a large volume of blood sample (up to 2ml/withdrawal)
- The animal is made comfortable in a restrainer while maintaining the temperature around at 24 to 27°C.
- The tail should not be rubbed from the base to the tip as it will result in leukocytosis. If the vein is not visible, the tail is dipped into warm water (40°C).
- Local aesthetic cream must be applied on the surface of the tail 30 min before the experiment.
- A 23G needle is inserted into the blood vessel and blood is collected using a capillary tube or a syringe with a needle. In case of difficulties, 0.5 to 1 cm of surface of the skin is cut open and the vein is pricked with bleeding lancet or needle and blood is collected with a capillary tube or a syringe with a needle.
- Having completed blood collection, pressure/silver nitrate ointment/solution is applied to stop the bleeding.
- If multiple samples are needed, temporary surgical cannula may be used.
- Restrainer is washed frequently to avoid/prevent pheromonally induced stress or cross infection [Figure 2].

## PROCEDURE FOR TAIL SNIP BLOOD SAMPLE COLLECTION

Requirements include animal, anesthetic agent, cotton, surgical blade and blood sample collection tubes.

- This method is recommended for blood collection only in mice.
- This method should be avoided as far as possible because it can cause potential permanent damage on the animal tail. If needed, it should be done under terminal anesthesia only.
- Before collecting the blood, local anesthesia is applied on the tail and a cut is made 1 mm from the tip of the tail using scalpel blade.
- Blood flow is stopped by dabbing the tail tip.

## PROCEDURE FOR ORBITAL SINUS BLOOD SAMPLE COLLECTION

Requirements include animal, anesthetic agent, cotton, capillary tube and blood sample collection tubes.

**Figure 3:**
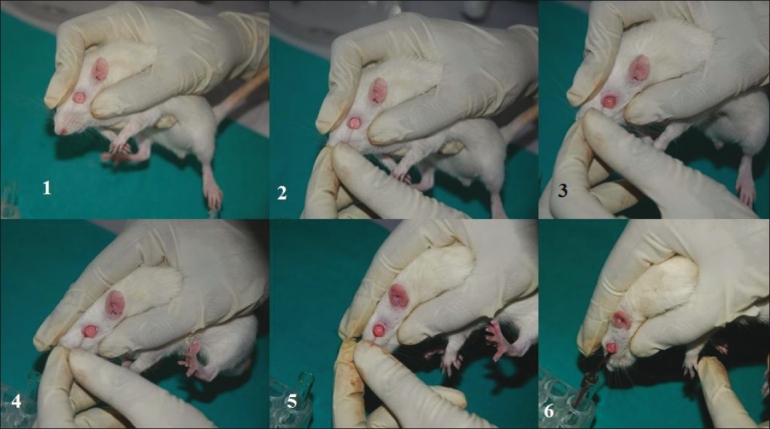
Blood sample collection from rat orbital sinus

**Figure 4:**
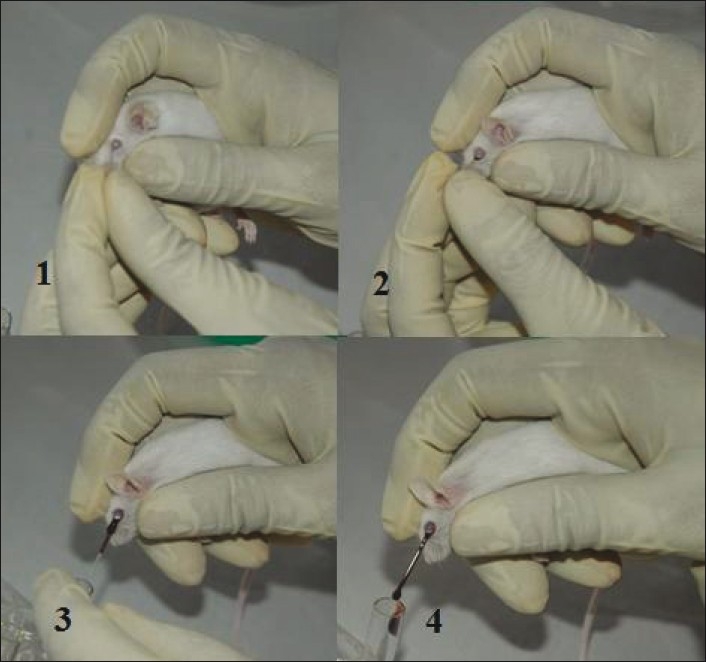
Blood sample collection from mouse orbital sinus

- This technique is used with recovery in experimental circumstances and this method is also called periorbital, posterior-orbital and orbital venous plexus bleeding.
- Blood sample is collected under general anesthesia.
- Topical ophthalmic anesthetic agent is applied to the eye before bleeding.
- The animal is scruffed with thumb and forefinger of the nondominant hand and the skin around the eye is pulled taut.
- A capillary is inserted into the medial canthus of the eye (30 degree angle to the nose).
- Slight thumb pressure is enough to puncture the tissue and enter the plexus/sinus.
- Once the plexus/sinus is punctured, blood will come through the capillary tube.
- Once the required volume of blood is collected from plexus, the capillary tube is gently removed and wiped with sterile cotton. Bleeding can be stopped by applying gentle finger pressure.
- Thirty minutes after blood collection, animal is checked for postoperative and periorbital lesions [Figures 3 and 4].
- Caution:
  - Repeated blood sampling is not recommended.
  - Skill is required to collect blood.
  - Even a minor mistake will cause damage to the eyes.
  - Two weeks should be allowed between two bleedings.
- Adverse effects reported from this method is around 1 to 2% which includes hematoma, corneal ulceration, keratitis, pannus formation, rupture of the globe, damage of the optic nerve and other intraorbital structures and necrotic dacryoadenitis of the harderian gland.

## PROCEDURE FOR JUGULAR VEIN BLOOD SAMPLE COLLECTION

Requirements include animal, anesthetic agent, cotton, 25G needle and blood sample collection tubes.

- In this method, warming of the animals is not required and is used to collect micro volumes to one ml of blood sample.
- This method has to be carried out under general/inhalation anesthesia and two persons are needed to collect blood sample.
- One person has to restrain the animal and monitor the animal. Another person is required to collect the blood sample from the animal.
- The neck region of the animal is shaved and kept in hyperextended position. The jugular veins appear blue in color and is found 2 to 4 mm lateral to sternoclavicular junction. A 25G needle is inserted in the caudocephalic direction (back to front) and blood is withdrawn slowly to avoid collapse of these small blood vessels. Animal has to be handled carefully and not more than 3 to 4 mm of needle is to be inserted into the blood vessel.
- If the attempt to collect blood fails, the needle is slowly removed and the site is monitored for bleeding. If there is no bleeding, one more attempt can be made. Further attempts should be avoided in case of bleeding as it may collapse the vein.
- Finger pressure is applied to stop bleeding.
- Caution:
  - Number of attempts is limited to three.
  - Apply local anesthetic cream 30 minutes prior to sampling.

## PROCEDURE FOR BLOOD SAMPLE COLLECTION WITH TEMPORARY CANNULA

Requirements include animal, anesthetic agent, cotton, 25G needle, animal warming chamber and blood sample collection tubes.

- Usually a temporary cannulation is made in the tail vein and used for a few hours.
- The animal is restrained and local anesthetic cream is applied on the tail (1 – 2 cm above the tail tip).
- The tail is either cannulated or a 25G needle is used.
- Tail bleeding normally requires the animal to be warmed in order to dilate the blood vessels (37 – 39°C for 5 – 15 min).
- After cannulation, animal has to be housed individually in large cages.

## PROTOCOL FOR BLOOD VESSEL CANNULATION

Requirements include animal, anesthetic agent, cotton, 25G needle, i.v. cannula, surgical blade, heparin (or any anticoagulant) and blood sample collection tubes.

**Figure 5:**
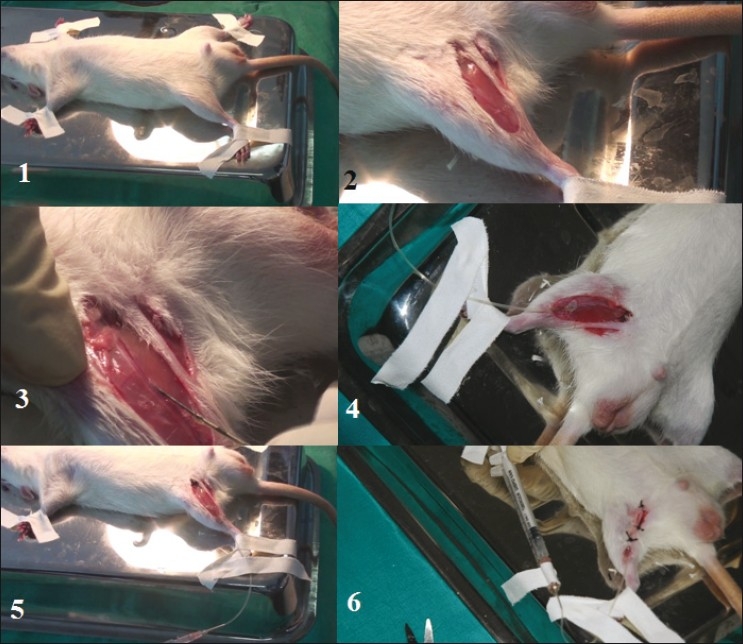
Blood vessel cannulation of rat femoral vein

- This method involves continuous and multiple sampling in the experimental animal.
- This method requires close and continuous monitoring of the animal.
- Usually blood vessel cannulation is done in the femoral artery, femoral vein, carotid artery, jugular vein, vena cava and dorsal aorta.
- Surgery is required for this method and appropriate anesthesia and analgesia should be used to minimize the pain.
- After surgical cannulation, animal should be housed singly in a large and spacious cage.
- Blood sample may be collected over 24 hour at the volume of 0.1 to 0.2 ml/sample.
- After withdrawing the blood, the cannula is flushed with an anticoagulant and the withdrawn volume may be replaced (if required) with LRS and cannula should be closed tightly [Figure 5].
- Caution: The experiment has to be conducted fully under aseptic precautions. Infection, hemorrhage, blockage of cannula and swelling around the cannulation site should be looked for. The needle size and maximum blood volume to be collected are given in Table 2.

**Table 2:** Needle size used for blood vessel cannulation in different species

Species	Needle to be used	Maximum collection volume
Mice	23 – 25G	1 ml
Rat	19 – 21G	10 – 15 ml
Rabbit	19 – 21G	60 – 200 ml
Guinea pig	20 – 21G	1 – 25 ml

## PROTOCOL FOR TARSAL VEIN BLOOD SAMPLE COLLECTION

Requirements include animal, anesthetic agent, cotton, 22G needle, hair remover and blood sample collection tubes.

**Figure 6:**
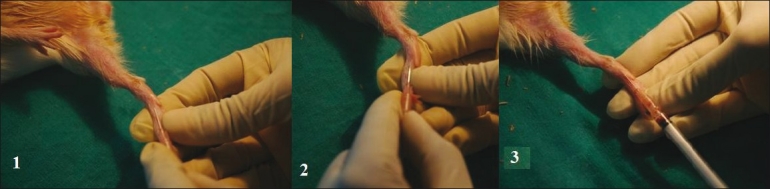
Blood sample collection from guinea pig tarsal vein

- Tarsal vein is identified in one of the hind legs of large animals. This method is commonly recommended for guinea pig.
- One person has to restrain the animal properly. Tarsal vein may be visible in blue color.
- The surface hairs are removed by applying a suitable hair remover. A local anesthetic cream is applied on the collection site.
- After 20 to 30 minutes, blood sample is collected slowly by using 22G needle.
- Maximum three samples can be taken per leg and 0.1 to 0.3 ml of blood can be collected per sample.
- After the sample collection, gentle pressure is applied with finger for 2 minutes to stop bleeding [Figure 6].
- Caution:
  - Not more than six samples from both hind legs are taken.
  - The number of attempts is three or less.

## PROTOCOL FOR MARGINAL EAR VEIN/ARTERY BLOOD SAMPLE COLLECTION

Requirements include animal, anesthetic agent, cotton, 26G needle, 95% v/v alcohol, o-Xylene, surgical blade and blood sample collection tube.

**Figure 7:**
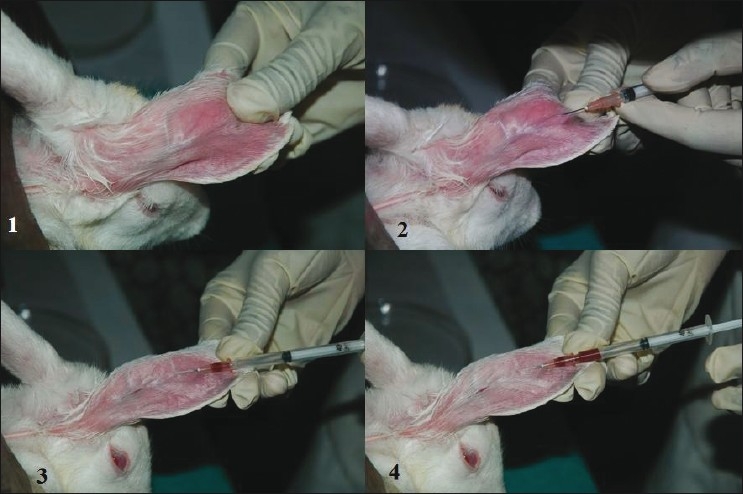
Blood sample collection from rabbit marginal ear vein using 26 G needle

**Figure 8:**
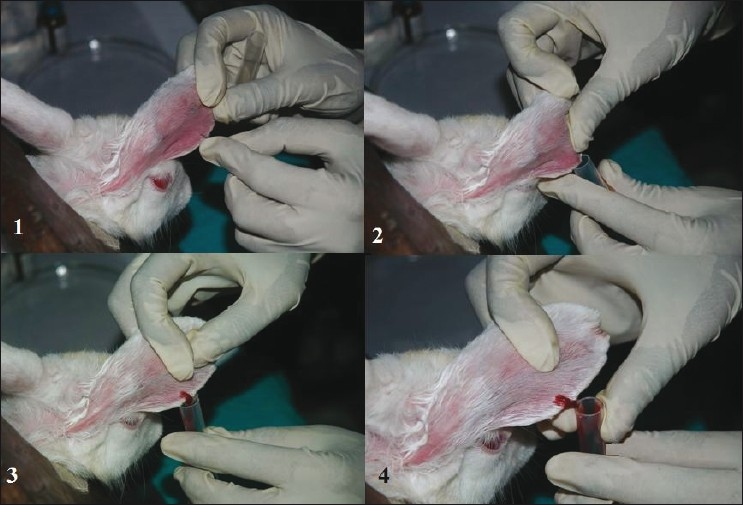
Blood sample collection from rabbit marginal ear vein using incision method.

- This method is commonly adopted for rabbits.
- The animal should be placed in a restrainer.
- Ear is cleaned with 95% v/v alcohol and local anesthetic cream is applied on the collection site 10 min prior to sampling. (If required, the o-Xylene/topical vasodilator may be applied topically on the collection site to dilate blood vessels).
- Size 11 surgical blade is used to cut the marginal ear vein and blood is collected in a collecting tube. Otherwise, a 26G needle may be used to collect blood from animal marginal vein.
- After collecting blood, clean sterile cotton is kept on the collection site and finger pressure is applied to stop the bleeding [Figures 7 and 8].

## PROTOCOL FOR CARDIAC PUNCTURE[10,11]

Requirements include animal, anesthetic agent, towel, cotton, 19 to 25G needle with 1 to 5 ml syringe, surgical blade, tube (internal diameter of 0.1 to 0.3 mm) for thoracotomy, plastic disposable bag and blood sample collection tubes.

**Figure 9:**
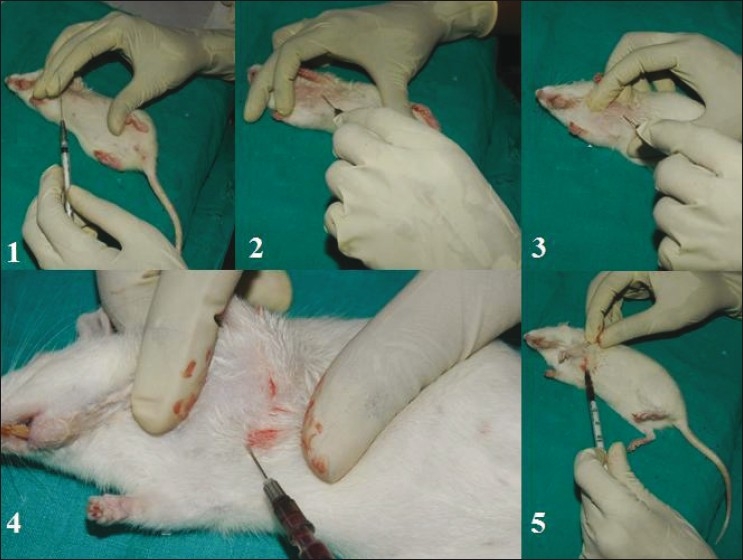
Blood sample collection through cardiac puncture in rat

- In general, cardiac puncture is recommended for terminal stage of the study to collect a single, good quality and large volume of blood from the experimental animals.
- During blood sample collection, animal will be in terminal anesthesia.
- Appropriate needle is used for blood sample collection with or without thoracotomy. Blood sample will be taken from the heart, preferably from the ventricle slowly to avoid collapsing of heart [Figure 9].
- Caution: If animal has dextrocardia, sampling may fail.

## PROTOCOL FOR BLOOD SAMPLE COLLECTION THROUGH POSTERIOR VENA CAVA

Requirements include animal, anesthetic agent, surgical blade, small glass rods, surgical scissor, 21 to 25G needle with 1 to 5 ml syringe and blood sample collection tube.

- In general, posterior vena cava blood sample is recommended for terminal stage of the study.
- Animal have to be anesthetized and ‘Y’- or ‘V’-shaped cut in the abdomen is made and the intestines are gently removed.
- The liver is pushed forward and the posterior vena cava (between the kidneys) is identified.
- 21 to 25G needle is inserted to collect blood from the posterior vena cava.
- This procedure will be repeated three to four times to collect more volume of blood sample.

## DISCUSSION

Blood collection from the experimental animals is one of the important procedures in biomedical research. Even a small error in the collection procedure may lead to a lot of variation in the results.

### Points to be remembered

- Before starting any kind of blood sample collection, it must be ensured that all chemical, surgical, fluid requirements are available in the working site.
- Not more than two to three attempts should be made to collect any kind of *in vitro* biological sample (excluding biological secretion).
- The blood collection tube must be labeled before starting the experiment and blood sample collected in the appropriately labeled collection tube.
